# Retroperitoneal multiple giant liposarcoma: a case report

**DOI:** 10.3389/fsurg.2025.1728768

**Published:** 2025-12-18

**Authors:** Jiaxin Hou, Qingqiang Yang

**Affiliations:** Department of Gastrointestinal, Hernia and Abdominal Wall Surgery, The Affiliated Hospital of Southwest Medical University, Luzhou, China

**Keywords:** retroperitoneal liposarcoma, huge volume, diagnosis, R0 resection, case report

## Abstract

The retroperitoneal anatomical space is located below the diaphragm and above the pelvic diaphragm, in the potential space between the posterior parietal peritoneum and the abdominal transverse fascia. Retroperitoneal liposarcoma is a common tumor in this region of the body. It typically presents no obvious clinical symptoms in the early stage due to the capaciousness of the area. Usually, however, an increase in tumor volume causes compression of the surrounding tissues and organs, such as intestinal obstruction and urinary obstruction, which leads to prominent symptoms. Primary giant retroperitoneal liposarcoma is a rare clinical entity. A comprehensive review of the literature reveals only a limited number of documented cases, with heterogeneity in their presentation, management, and outcomes. This report presents a case of multiple giant retroperitoneal liposarcomas successfully managed with multivisceral resection achieving R0 status. This case highlights the surgical challenges and feasibility of complete resection even in massive and multifocal tumors.

## Introduction

Retroperitoneal liposarcoma (RPLS) is the most common primary malignancy of the retroperitoneal space, characterized by a high rate of local recurrence and a propensity for significant growth before clinical detection (1). Achieving a complete (R0) surgical resection is the cornerstone of curative therapy and is the most significant predictor of long-term survival (2). However, because of the anatomical complexity of the retroperitoneum and the frequent involvement of adjacent organs, obtaining negative margins often necessitates complex multivisceral resections (3). The surgical management of giant (variably defined as >20–30 cm) and multifocal RPLS presents a formidable challenge to even experienced surgical oncologists. Although R0 resection rates for RPLS have been documented in the literature, detailed reports on the successful *en bloc* resection of multiple (4) giant synchronous tumors are scarce. Publishing such cases is critical for several reasons. First, this study contributes to medical knowledge by providing a real-world template for surgical strategy, operative planning, and perioperative management for exceptionally complex cases. Second, it has a direct impact on patient care by demonstrating that curative-intent surgery is feasible in scenarios that might otherwise be deemed inoperable, thereby setting a benchmark for outcomes and inspiring similar efforts in well-selected patients. Finally, this case holds relevance for current clinical practice, where multidisciplinary team (MDT) approaches are increasingly emphasized. It serves as a valuable reference for discussions within MDTs regarding the extent of safe resection, the balance between radicality and morbidity, and the importance of centralized care for rare and complex sarcomas.

## Case introduction

**Figure 1:**
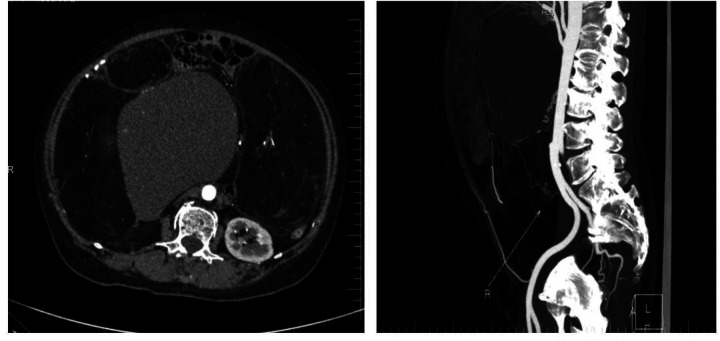
Contrast-enhanced CT.

**Figure 2:**
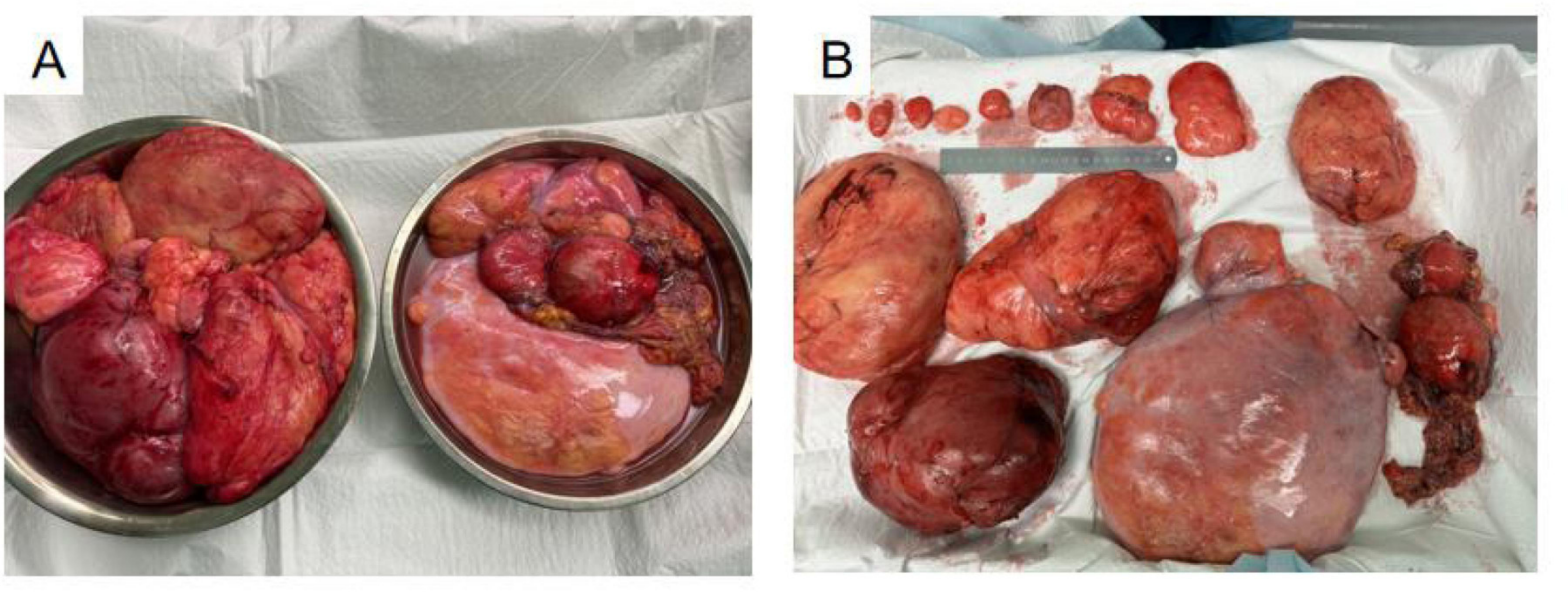
**(A,B)** Tumor pattern after complete resection.

**Figure 3:**
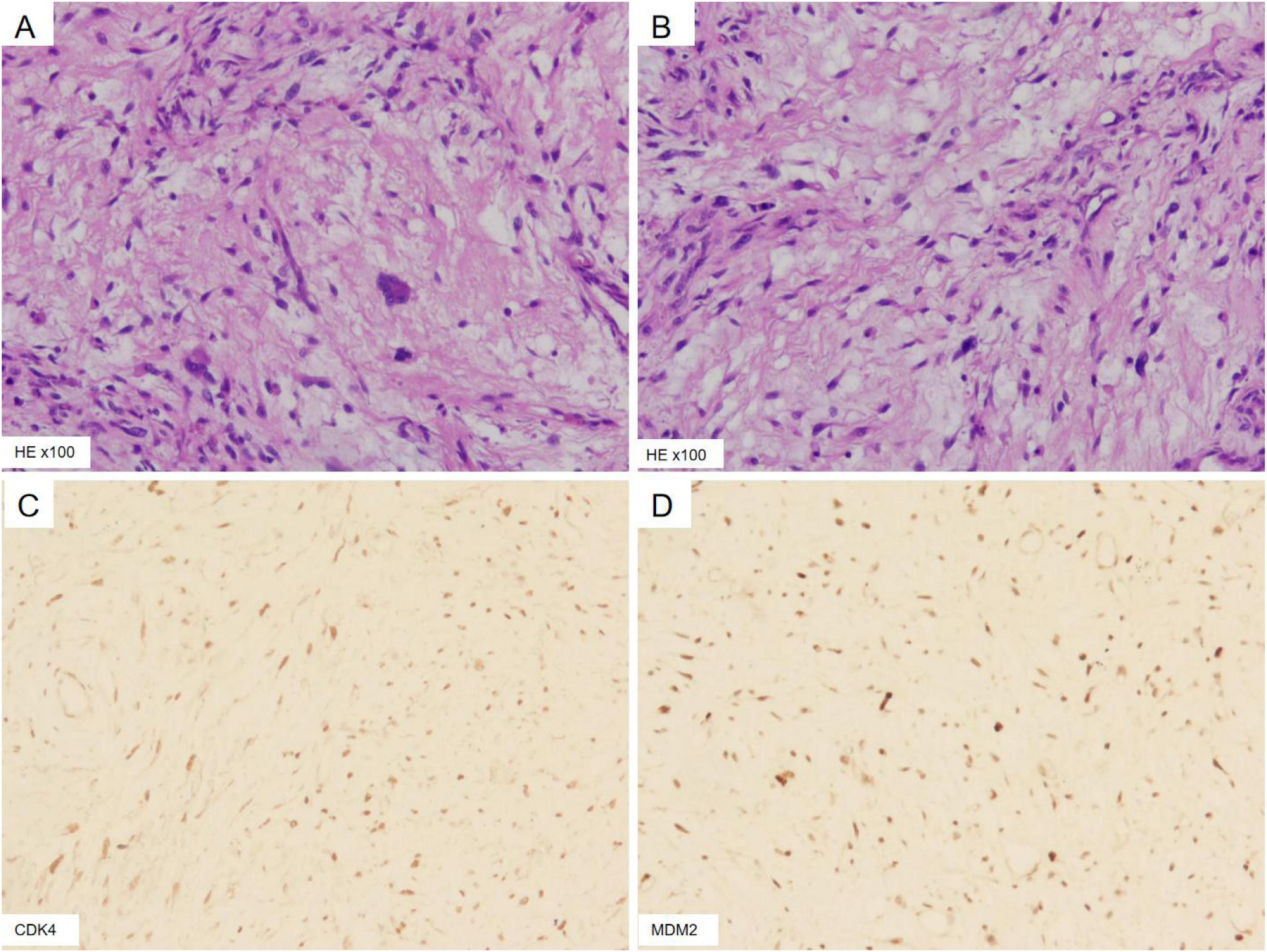
**(A,B)** Postoperative pathological examination and **(C,D)** related immunohistochemical examination.

As summarized in Table 1, this report presents a case of multiple giant retroperitoneal liposarcomas successfully managed with multivisceral resection achieving R0 status. A 57-year-old woman was admitted to the hospital for abdominal distension over a course of 2 months, mainly manifesting as abdominal distension and discomfort with changes in defecation habits. She had no obvious abdominal pain, nausea, dyspnea, or other symptoms. Her past medical history was unremarkable. On physical examination, there was full abdominal swelling. There was a diffuse tangible mass, with an unclear boundary. Relevant auxiliary examinations were improved. Abdominal enhanced CTA revealed a large mixed-density shadow in the abdominal cavity, the boundary of which was unclear, of approximately 28.9 cm × 17.0 cm × 29.6 cm, (Figure 1), with visible solid and fat components. The adjacent intestine, pancreas, blood vessels, uterus, and bladder were pushed, displaced, and deformed. The abdominal tumor was sizable, compressing the surrounding tissues and organs. In view of the large tumor and the unclear boundary with the surrounding tissues, a dedicated MDT meeting was convened prior to surgery to review this complex case. The panel included surgical oncologists specializing in soft-tissue sarcoma, a radiologist with expertise in abdominal imaging, a pathologist, and a medical oncologist. The discussion centered on the feasibility of achieving a complete (R0) resection given the massive size and multifocal nature of the tumor. Based on preoperative imaging, the team unanimously agreed on a diagnosis of primary retroperitoneal liposarcoma. After careful consideration of the risks and benefits, the team reached a consensus that proceeding with an *en bloc* multivisceral resection with curative intent was the most appropriate management strategy. The surgical plan, including the potential need for resection of the adjacent organs (e.g., intestine), was outlined and approved by the MDT. The patient underwent gastrointestinal surgery following consent from her family. During the operation, the abdominal cavity was opened and multiple tumors were identified. Multiple tumors were observed in the greater omentum. The largest tumor was approximately 40 cm × 20 cm, and consisted of soft, yellow, fat-like tissue. A smaller hard tumor of approximately 8 cm × 6 cm was also observed. Multiple giant retroperitoneal tumors were located behind the mesocolon, up to the upper border of the pancreas, down to the lower part of the sacrum, and on both sides on the outside of the psoas major. Each solitary tumor was surrounded by fibrous hard tissue, with the tumor tissue being adipose. Moreover, the left reproductive blood vessel passed through the left retroperitoneal lipoma. The right hemicolon and small intestine were displaced to the left upper abdomen. Multiple yellow, soft, fat-like tumor tissues were observed in the ascending mesocolon, with the largest measuring approximately 10 cm × 5 cm. Moreover, a yellow, soft, fat-like tumor tissue measuring about 20 m × 1.5 cm was seen in the small intestine, located approximately 160 cm from the ileocecal valve. The operation was successfully completed, and multiple tumors were completely removed from the abdominal cavity (Figure 2). Postoperatively, the patient received standard care, including electrocardiographic monitoring, supplemental oxygen, and fluid resuscitation. Her recovery course was uneventful, and the patient was discharged on the sixth postoperative day. Histopathological examination of the resected specimen was performed. According to the 2020 World Health Organization (WHO) classification of soft tissue tumors (5), the final diagnosis was confirmed as well-differentiated liposarcoma (WDLPS) (Figure 3). TNMG staging was stage III (T4N0M0 and G1). The capsules of multiple tumors were intact, with no positive margin. R0 resection was confirmed. Immunohistochemical analysis provided the following results: CK (−), Vim (+), MDM2 (+), CDK4 (+), RB1 (deletion), P16 (+), S100 (−), C034 (−), P53 (wild type), and Ki67 (+, 10%). At 6 months postoperatively, the patient underwent reexamination with an abdominal CT scan and necessary laboratory tests.

**Table 1:** Literature review of reported cases of giant retroperitoneal liposarcoma.

First author, year	Sex/age	Tumor size (cm)	Primary treatment
Yol, 1998 (18)	M/63	35	Surgical resection
McCallum, 2006 (19)	F/47	50 × 48 × 45	Surgical resection
Clar, 2009 (20)	M/66	47 × 25 × 42	Surgical resection
Hashimoto, 2010 (21)	M/41	30 × 30	Surgical resection
Bansal, 2013 (22)	M/52	40 × 35 × 35	Surgical resection
De Nardi, 2012 (23)	M/40	50 × 49 × 35	Surgical resection
Sharma, 2013 (24)	F/60	40 × 40 × 25	Surgical resection
Zhang, 2015 (2)	F/48	20 × 15	Surgical resection
Caizzone, 2015 (25)	F/64	42 × 37 × 18	Surgical resection
Hazen, 2017 (26)	M/64	40	Surgical resection
Oh, 2016 (27)	F/71	45 × 30 × 15	Surgical resection
Zeng, 2017 (28)	M/45	65 × 45 × 30	Surgical resection
Herzberg, 2019 (29)	F/75	35 × 29 × 20.5	Surgical resection
Xu, 2020 (30)	M/65	37 × 32 × 26.5	Surgical resection
Spicer, 2021 (31)	M/37	31	Surgical resection
Herrera-Almario, 2022 (32)	M/55	70 × 50 × 10	Surgical resection
Suryabanshi, 2022 (33)	M/62	30 × 28 × 21	Surgical resection
Ye, 2022 (34)	M/54	32 × 21 × 12	Surgical resection
Xia, 2022 (35)	F/50	45 × 30 × 20	Surgical resection
Liu, 2022 (36)	M/70	33 × 35 × 28	Surgical resection
Mansour 2022 (37)	M/33	50	Surgical resection
Lieto, 2022 (38)	M/61	70	Surgical resection
Trajkovski, 2022 (39)	F/66	56 × 52 × 20	Surgical resection
Evola, 2022 (40)	F/55	36 × 32 × 28	Surgical resection
Wei, 2022 (41)	F/51	32	Surgical resection
Rachman, 2022 (42)	F/34	28 × 32	Surgical resection
Tani, 2022 (43)	F/78	25 × 20	Surgical resection
Chen, 2022 (44)	M/68	38 × 28 × 18	Surgical resection
Luke, 2022 (45)	M/49	30 × 32 × 15	Surgical resection
Cheng, 2023 (46)	M/55	44.5	Surgical resection
Habonimana, 2023 (47)	M/58	30 × 25 × 8	Surgical resection
Gutu, 2023 (48)	F/63	27 × 29 × 36	Surgical resection
Tripathi, 2023 (49)	M/57	66 × 38 × 37	Surgical resection
Diaz, 2013 (50)	M/41	33 × 31 × 29	Surgical resection
Jia-Ning Sun 2024 (3)	M/58	55 × 30 × 18	Surgical resection
Ren Yingzheng 2025 (51)	F/55	74 × 54 × 24	Surgical resection
Current Case, 2025	M/57	28.9 × 17.0 × 29.6	Surgical resection

## Discussion

RPLS is the most common primary malignancy of the retroperitoneal space, accounting for approximately 45% of such tumors (6). Owing to its deep and concealed anatomical location, RPLS often attains a considerable size before clinical detection, typically presenting with symptoms caused by the compression of adjacent tissues and organs (7). Complete surgical resection remains the cornerstone of curative treatment. Achieving an R0 resection during the primary surgery represents the most critical opportunity for a potential cure and is the most significant prognostic factor that can be influenced by surgical intervention (8). Preoperative evaluation is crucial; however, the frequent involvement of surrounding structures by these massive tumors often necessitates complex multivisceral resections, with surgical difficulty and risk escalating proportionally with increasing tumor size (9).

In the present case, an R0 resection was successfully achieved without administering preoperative or postoperative radiotherapy or chemotherapy. This decision was reached through multidisciplinary discussion and reflects the current nuanced evidence. While some studies, such as the recent report by Baudo et al. (10), suggest potential survival benefits for perioperative radiotherapy in non-metastatic RPS, aligning with trends in National Comprehensive Cancer Network (NCCN) guidelines (11), its absolute benefit—particularly for the WDLPS subtype present in our patient—remains a subject of ongoing debate, as highlighted by the STRASS trial (12). Given the massive size and complex anatomy, preoperative radiotherapy posed potential risks of delaying surgery and increasing technical complications. Furthermore, the well-differentiated histology is widely recognized to be largely insensitive to conventional chemotherapy (13). Therefore, our strategy prioritized maximizing the success of the initial surgical resection. The patient's disease-free status at 12 months following surgery is encouraging. In comparison with other studies, patients with WDLPS who undergo R0 resection typically demonstrate a favorable prognosis, with median disease-free survival (DFS) extending over several years and favorable 5-year overall survival (OS) rates (12). These findings underscore the paramount importance of achieving a radical resection. From this case, we reaffirm that high-quality preoperative imaging and early involvement of a multidisciplinary team (MDT) are indispensable for planning complex, curative-intent surgery. The preoperative MDT consensus on the need for *en bloc* resection was instrumental in achieving this favorable outcome (52). However, we must acknowledge the limitations inherent in this report. First, this is a single case report, which is descriptive in nature and therefore cannot establish causality or provide generalizable results. Second, the follow-up duration remains relatively short, limiting our ability to assess long-term OS and DFS. Therefore, continued close surveillance is essential. Moreover, the immunohistochemical findings in our case, confirming the diagnosis of WDLPS, also provided insight into the molecular underpinnings of this disease. Well-differentiated and dedifferentiated liposarcomas are characterized by supernumerary ring and giant marker chromosomes containing amplified sequences of the *12q13-15* region, which harbors key oncogenes such as CDK4 and MDM2 (14). This distinct molecular signature provides a compelling rationale for exploring targeted therapies, which aim to overcome the limitations of conventional radiotherapy and chemotherapy. For instance, CDK4/CDK6 inhibitors (e.g., palbociclib) and MDM2 antagonists are under active investigation in clinical trials for advanced liposarcoma (15). Although our patient does not currently require adjuvant therapy, the identification of these molecular targets provides a valuable strategic option in the event of future recurrence, when surgical reresection may not be feasible. Therefore, alongside surgical innovation, the integration of molecular profiling into the diagnostic workup of RPLS is becoming increasingly critical for personalizing treatment and improving long-term outcomes.

Even after complete resection, patients with RPLS remain at high risk of local recurrence, for which early surgical intervention is the primary treatment (16). However, reoperation is associated with significantly increased complexity and risk compared to the initial operation (17), further highlighting the critical window of opportunity provided by the first surgery. Future prospective studies and longer-term data are needed to refine multimodal management strategies for these challenging tumors.

## Conclusion

Giant retroperitoneal liposarcoma is a rare tumor disease. At present, preoperative diagnosis primarily depends on CT and MRI. However, it is through the postoperative pathological results that the disease is definitively diagnosed (6). Although the current treatment method is R0 surgical resection, such conditions are often discovered at an advanced stage, presenting with large tumors and invasion of surrounding tissues and organs, making surgery challenging. Multidisciplinary teamwork is therefore frequently required to achieve successful results (17).

## Data Availability

The original contributions presented in the study are included in the article/Supplementary Material, further inquiries can be directed to the corresponding authors.
